# Lethality of inappropriate plasma exposure on chicken embryonic development

**DOI:** 10.18632/oncotarget.21105

**Published:** 2017-09-20

**Authors:** Jiao Jiao Zhang, Jin Oh Jo, Do Luong Huynh, Mrinmoy Ghosh, Nameun Kim, Sang Baek Lee, Hak Kyo Lee, Young Sun Mok, Taeho Kwon, Dong Kee Jeong

**Affiliations:** ^1^ Department of Animal Biotechnology and Advance Next Generation Convergence Technology, Jeju National University, Jeju, Republic of Korea; ^2^ Department of Chemical and Biological Engineering, Jeju National University, Jeju, Republic of Korea; ^3^ Department of Animal Biotechnology, Chonbuk National University, Jeonju, Republic of Korea; ^4^ Laboratory of Animal Genetic Engineering and Stem Cell Biology, Subtropical/Tropical Organism Gene Bank, Jeju National University, Jeju, Republic of Korea

**Keywords:** non-thermal DBD plasma, chicken embryo, ROS, NRF2, ATP

## Abstract

In this study, we examined the effects of non-thermal dielectric barrier discharge plasma on embryonic development in chicken eggs in order to determine the optimal level of plasma exposure for the promotion of embryonic growth. We exposed developing chicken embryos at either Hamburger-Hamilton (HH) stage 04 or HH 20 to plasma at voltages of 11.7 kV to 27.6 kV. Our results show exposure at 11.7 kV for 1 min promoted chicken embryonic development, but exposure to more duration and intensity of plasma resulted in dose-dependent embryonic death and HH 20 stage embryos survive longer than those at stage HH 04. Furthermore, plasma exposure for 4 min increased the production of reactive oxygen species (ROS) and inactivated the nuclear factor erythroid 2-related factor 2 (NRF2)-antioxidant response signaling pathway, resulting in suppression of antioxidant enzymes in the skeletal muscle tissue of the dead embryos. We also found decreased levels of adenosine triphosphate production and reductions in the expression levels of several growth-related genes and proteins. These findings indicate that inappropriate plasma exposure causes dose-dependent embryonic death via excessive accumulation of ROS, NRF2-antioxidant signaling pathway disruption, and decreased growth factor expression.

## INTRODUCTION

Physical plasma is ionized gas consisting of charged particles, radicals, reactive atoms and molecules, and ultraviolet photons. Non-thermal dielectric barrier discharge (DBD) plasma is a type of plasma that occurs at atmospheric pressure when either high voltage time-varying waveform or short duration pulses are applied to two electrodes, creating electrically safe plasma without substantial gas heating [1–3]. Advancements in DBD plasma system with a discharge sufficiently uniform and moderate neutral gas close to room temperature promote its medical and biological applications to living cells and tissues [2, 4–8]. In this previous study, we found that exposure to plasma at specific potentials and durations promoted soybean seed germination and sprout growth, while excess exposure inhibited growth [9]. This study is inspired by our previous work examining the effects of non-thermal DBD plasma on the promotion of plant growth. In the study, we decided to determine whether non-thermal DBD plasma affects growth rate in animals. To answer this question, we examined the effects on embryonic development in chickens after irradiation of fertilized eggs with non-thermal DBD plasma.

Chickens have unique embryonic development characteristics, as a result of oviparity, which are easily accessible and can be manipulated without maternal impact or external influences. Embryonic development is observable via candling and the exact embryonic age can easily be determined based on size and appearance [10]. Furthermore, early cell division, growth, and segregation into specific tissues occurs even in unincubated fertilized eggs [11]. When incubation starts, embryonic development continues, the embryo contains all organs needed to sustain life after hatching and the majority of these organs can be easily identified by the end of Hamburger-Hamilton (HH) stage 20 [10]. During embryonic development, a stage-dependent reaction on chemical and physical factors was found in mammals [12]. Unlike mammals, chicken embryos have no preimplantation stages, but resistance of chicken embryos at different stages of development to various external forces [13] and an age-related cellular resistance to viral infections were also found during chicken embryogenesis [14]. Furthermore, various cells have been shown to be highly susceptible to plasma treatment [15–17]. Whether chicken embryos have sensitivity to plasma treatment and exist stage-dependent reactions remain to be answered.

*In vitro* experiments with fibroblasts [18], endothelial cells [1], epithelial cells [2], myoblast cells [19], and tumor cells [20] have demonstrated that the effects of plasma exposure on these cell types are dose-dependent. Low intensity plasma exposure enhanced endothelial and epithelial cell proliferation [1, 2], while high doses of plasma had anti-proliferative effects on various kinds of mammalian normal cells and cancer cells and induced apoptosis [18–20]. Therefore, we hypothesize that non-thermal DBD plasma affects chicken embryonic development in a dose-dependent manner during the early stages of development.

When cells or tissue surfaces are exposed to non-thermal DBD plasma a variety of biologically active reactive species are generated, particularly, reactive oxygen species (ROS) [3, 21, 22], which are known to directly activate multiple proteins involved in the signaling pathways that increase cell function [20, 23, 24]. ROS levels are controlled by an inducible antioxidant system that responds to cellular stressors and is predominantly regulated by nuclear factor erythroid 2-related factor 2 (NRF2) and its repressor protein kelch like ECH associated protein 1 (KEAP1) [25, 26]. NRF2-antioxidant signaling pathway is important for the amelioration of oxidative stress [27, 28]. Oxidative stress mediated the effects of non-thermal plasma on the inhibition of mammalian normal cells deficiencies [2, 29] and the synergistic apoptosis of malignant cells [20, 30] by increasing the formation of excessive intracellular ROS. Here, we examine the effects of non-thermal DBD plasma on chicken embryonic development and explore the roles of oxidative stress via mediating ROS level and NRF2-antioxidant signaling pathway.

## RESULTS

### Effect of plasma on embryonic development

**Figure 1:**
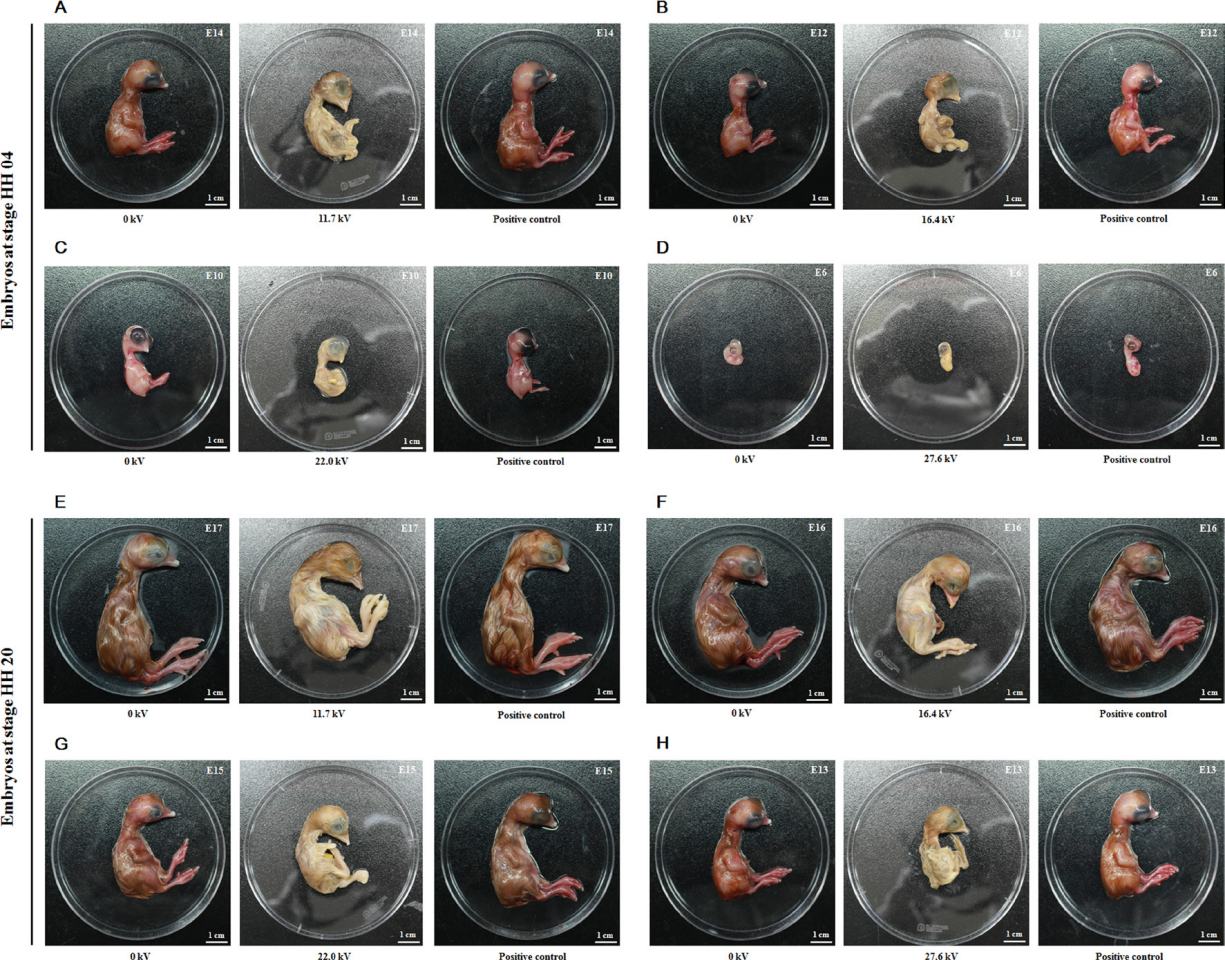
Effect of plasma on chicken embryonic development Embryos at stage HH 04 were treated at voltages of (**A**) 11.7 kV, (**B**) 16.4 kV, (**C**) 22.0 kV, (**D**) 27.6 kV and HH 20 were treated at voltages of (**E**) 11.7 kV, (**F**) 16.4 kV, (**G**) 22.0 kV, (**H**) 27.6 kV for 4 min, and returned to the incubator. Fertilized eggs not treated with plasma were used as the control group (0 kV). Embryos at stage HH 04 or HH 20 treated with plasma at 11.7 kV for 1 min were correspondingly used as the positive control. The death day was estimated according to the Hamburger-Hamilton stages. E represents the embryonic day.

All embryos from stages HH 04 and HH 20 died following treatment with non-thermal DBD plasma at voltages ranging from 11.7 kV to 27.6 kV for 4 min (Figure 1). As compared to the control embryos (no plasma treatment; 0 kV) and positive control embryos (stage HH 04 treated with plasma at 11.7 kV for 1 min), embryos at stage HH 04 exposed to 11.7 kV, 16.4 kV, 22.0 kV, and 27.6 kV plasma for 4 min were found to die, on average, at stages embryonic day 14 (E14), E12, E9.75, and E6.125, respectively, according to the Hamburger-Hamilton stages (Table 1, Figure 1A–1D) [10]. As compared to the control and positive control embryos (stage HH 20 treated with plasma at 11.7 kV for 1 min), embryos at stage HH 20 exposed to 11.7 kV, 16.4 kV, 22.0 kV, and 27.6 kV plasma for 4 min were found to die, on average, at stages E17, E16, E15, and E13, respectively (Table 1, Figure 1E–1H). These results show that exposure to plasma at higher voltages for 4 min increases the rate embryo death, and embryos at stage HH 20 survive longer than those at stage HH 04 which indicates that the stage HH 20 embryos are more resistant to the lethal effects of the plasma.

**Table 1:** Effect of plasma on chicken dead embryonic stage and number

Group	0 kV	Embryos at stage HH 04						Embryos at stage HH 20				Positive control
		11.7 kV	16.4 kV	22.0 kV		27.6 kV		11.7 kV	16.4 kV	22.0 kV	27.6 kV	
Dead embryonic stage	—	E14	E12	E10	E9	E7	E6	E17	E16	E15	E13	—
Dead number	0	8	8	6	2	1	7	8	8	8	8	0

Embryos at stage HH 04 or HH 20 were treated at voltages of 11.7 kV to 27.6 kV for 4 min, and returned to the incubator. Fertilized eggs not treated with plasma were used as the control group (0 kV). Embryos at stage HH 04 or HH 20 treated with plasma at 11.7 kV for 1 min were correspondingly used as the positive control. Eight fertilized eggs were used in each group. The number of dead embryos was recorded, and death day was estimated according to the Hamburger-Hamilton stages. E represents the embryonic day.

### Effect of plasma on ROS levels

**Figure 2:**
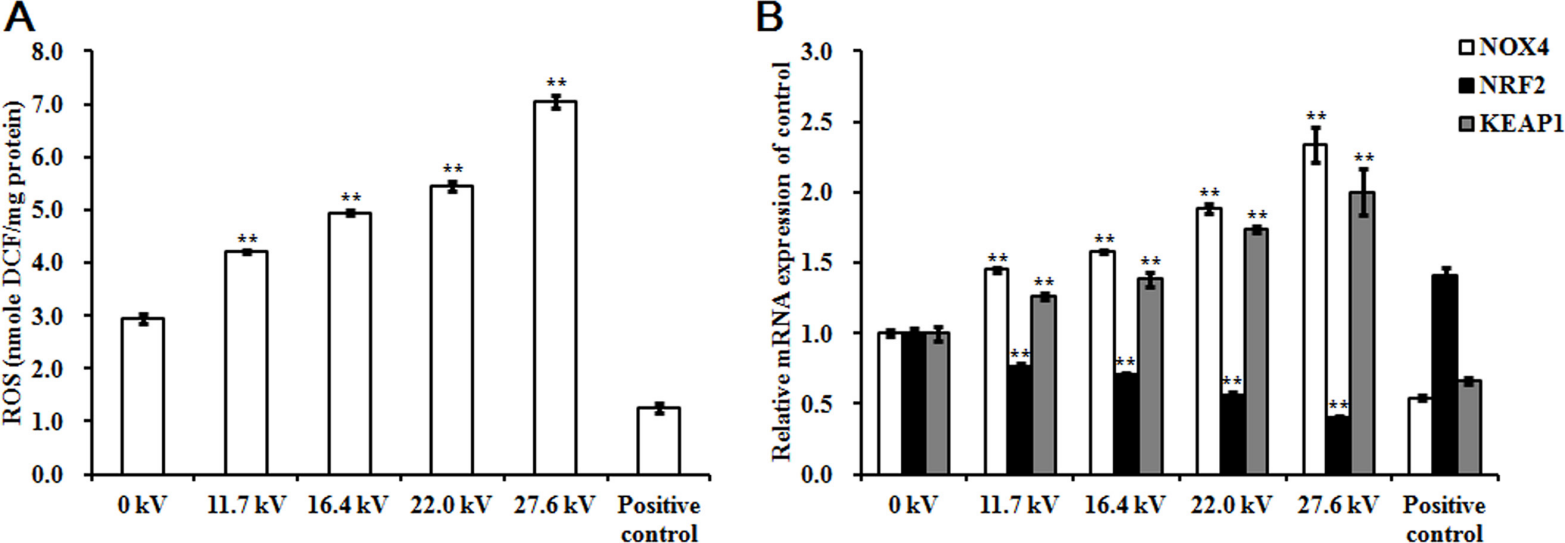
Effect of plasma on ROS levels (**A**) ROS levels, (**B**) Relative mRNA expression of *NOX4*, *NRF2*, and *KEAP1* in the skeletal muscle tissues of the chicken embryos at stage HH 20 exposed at different plasma potential for 4 min. Fertilized eggs not treated with plasma were used as the control group (0 kV). Embryos at stage HH 20 treated with plasma at 11.7 kV for 1 min were used as the positive control. ROS level in the skeletal muscle was expressed as nmole DCF/mg protein. mRNA expression was determined by RT-PCR analysis, and normalized to the *β-actin* mRNA level. Values are expressed as the mean ± standard error (*n* = 3). *p < 0.05 versus control; **p < 0.01 versus control, according to one-way ANOVA and LSD test.

ROS levels in the skeletal muscle tissues of the chicken embryos at stage HH 20 exposed to plasma for 4 min increased with increases in applied in electric potential as compared to the control and positive control groups. Exposure to 27.6 kV increased ROS levels by 1.40-fold (*p <* 0.001) over the control group (Figure 2A). Expression of nicotinamide adenine dinucleotide phosphate oxidase 4 (*NOX4*) mRNA expression, an enzyme that produces ROS, increased 1.34-fold (*p <* 0.001) following exposure to plasma at 27.6 kV for 4 min (Figure 2B). However, the mRNA expression level of *NRF2*, an activator of genes that encode enzymes and proteins involved in detoxification and antioxidant effects, decreased 0.60-fold (*p <* 0.001; Figure 2B) and protein levels decreased 0.38-fold (*p <* 0.001) after plasma treatment at 27.6 kV for 4 min (Figure 5A and 5B). Furthermore, the expression of *KEAP1*, which targets NRF2 for ubiquitination and subsequent proteolysis, increased 1.00-fold (*p <* 0.001) in mRNA expression (Figure 2B) and 0.82-fold (*p <* 0.001) in protein expression following plasma treatment at 27.6 kV as compared with the control group (Figure 5A and 5B).

### Antioxidant enzyme activity

**Figure 3:**
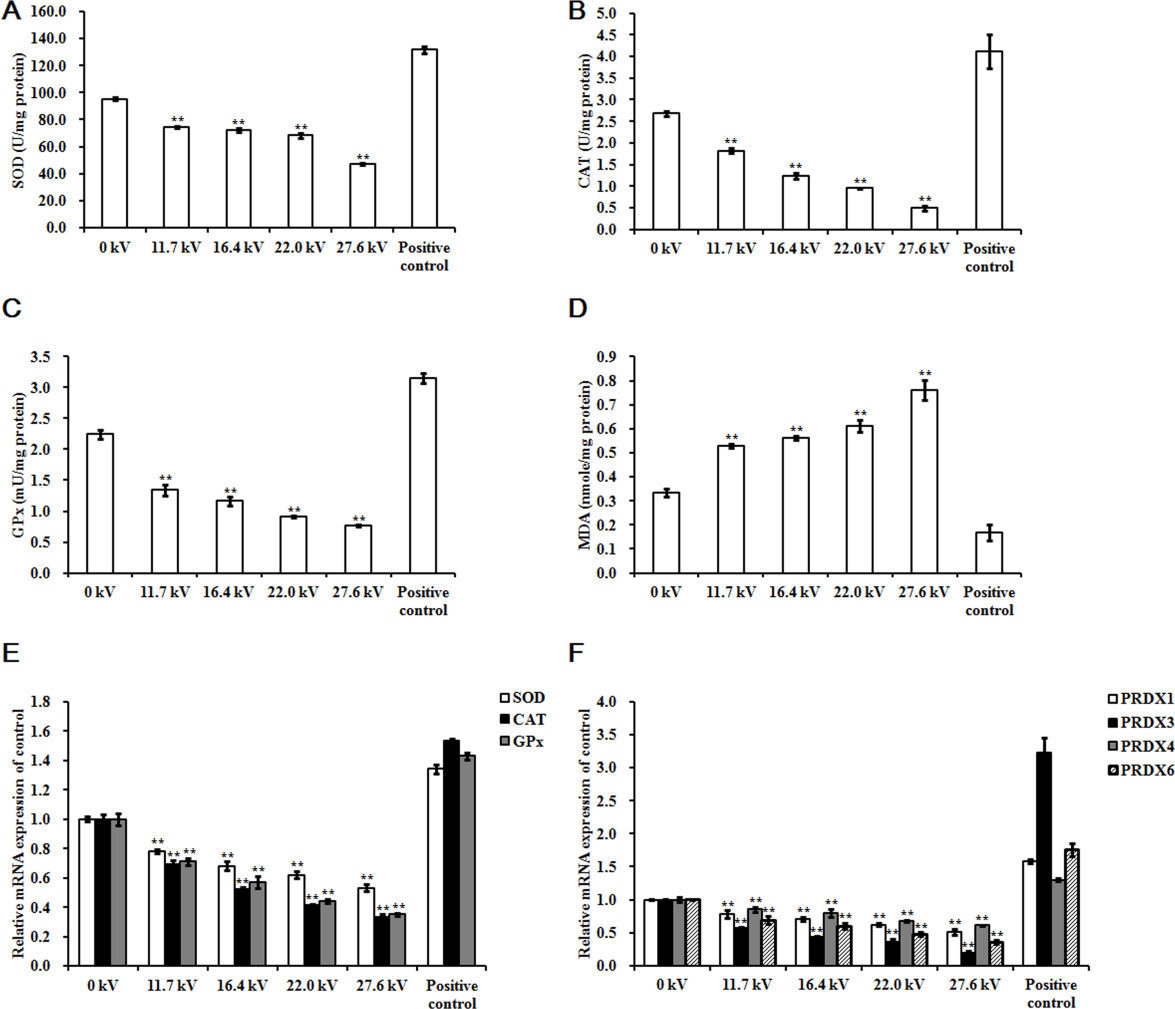
Effect of plasma on antioxidant enzyme Activities of (**A**) SOD, (**B**) CAT, (**C**) GPx, and (**D**) MDA, and relative mRNA expression of (**E**) *SOD*, *CAT*, and *GPx*, and (**F**) *PRDX1*, *PRDX3*, *PRDX4*, and *PRDX6* in the skeletal muscle tissues of the chicken embryos at stage HH 20 exposed at different plasma potential for 4 min. Control and positive control were as described before. mRNA expression was determined by RT-PCR analysis, and normalized to the *β-actin* mRNA level. Values are expressed as the mean ± standard error (*n* = 3). *p < 0.05 versus control; **p < 0.01 versus control, according to one-way ANOVA and LSD test.

The activities of the antioxidant enzymes superoxide dismutase (SOD), catalase (CAT), and glutathione peroxidase (GPx) decreased following exposure to plasma for 4 min in the skeletal muscle tissues of the dead embryos. However, malondialdehyde (MDA) levels increased in a dose-dependent manner. Treatments with 27.6 kV plasma elicited the greatest changes across the potential range (11.7 to 27.6 kV), resulting in 0.51- (*p <* 0.001), 0.82- (*p <* 0.001), and 0.66-fold (*p <* 0.001) decreases in SOD, CAT, and GPx, respectively, and a 1.29-fold (*p <* 0.001) increase in MDA (Figure 3A, 3B, 3C, and 3D). *SOD*, *CAT*, and *GPx* mRNA expression levels decreased following 4 min plasma exposure across the potential range (11.7 to 27.6 kV), in a dose-dependent manner, with the greatest decreases in *SOD*, *CAT*, and *GPx* of 0.47- (*p <* 0.001), 0.67- (*p <* 0.001), and 0.65-fold (*p <* 0.001) in the group treated with 27.6 kV, respectively (Figure 3E). The 27.6 kV plasma treatment had the largest inhibitory effect on peroxiredoxin (*PRDX*)*-*family gene expression in the skeletal muscle tissue of the dead embryos, resulting in decreases of 0.49- (*p <* 0.001), 0.80- (*p <* 0.001), 0.39- (*p <* 0.001), and 0.64-fold (*p <* 0.001) in *PRDX1*, *PRDX3*, *PRDX4*, and *PRDX6*, respectively (Figure 3F). PRDX3 protein expression decreased 0.68-fold (*p <* 0.001) after treatment at 27.6 kV for 4 min (Figure 5A and 5B).

### Levels of adenosine triphosphate (ATP) and growth-related factors

**Figure 4:**
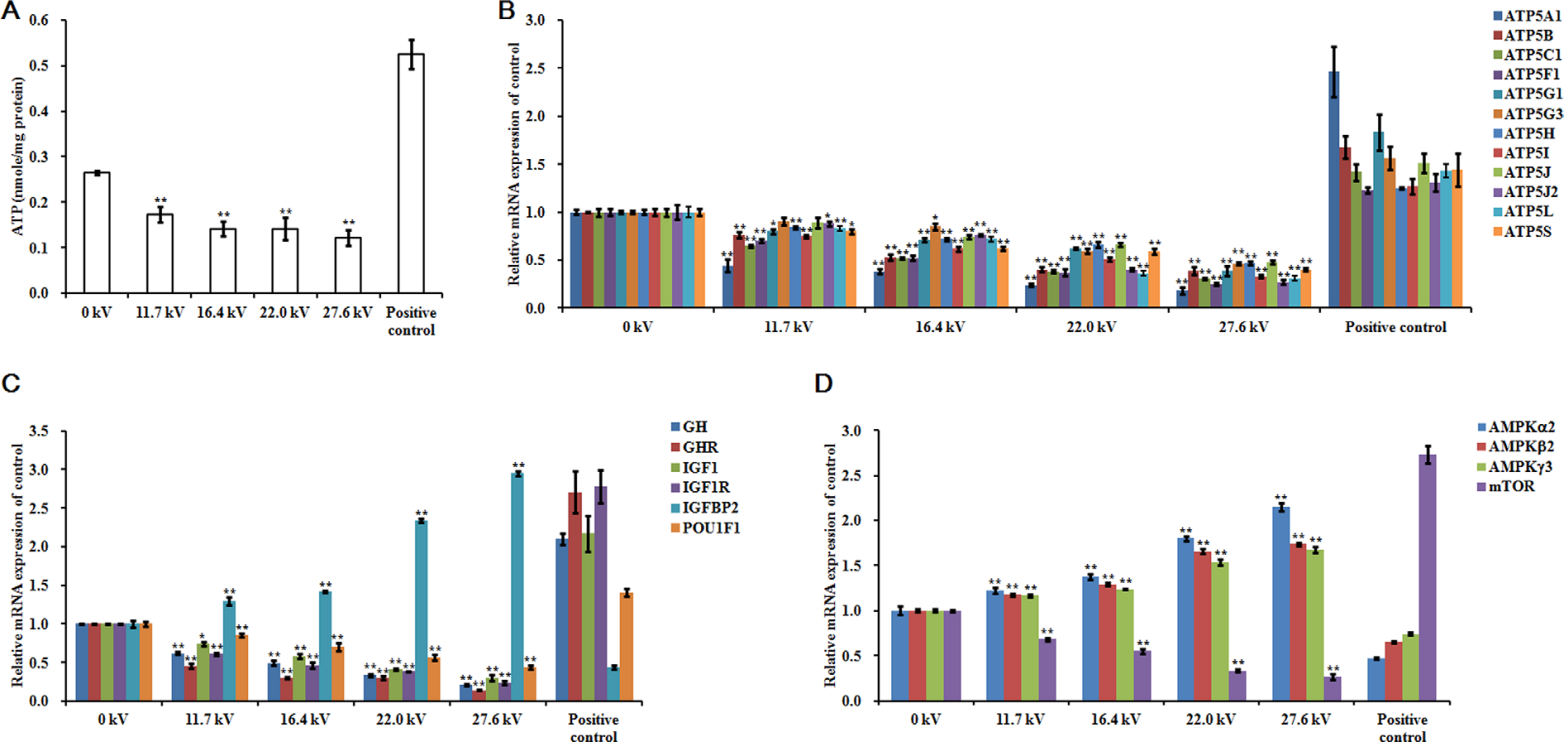
Effect of plasma on ATP and growth-related factors (**A**) ATP levels, relative mRNA expression of (**B**) *ATP5A1*, *ATP5B*, *ATP5C1*, *ATP5F1*, *ATP5G1*, *ATP5G3*, *ATP5H*, *ATP5I*, *ATP5J*, *ATP5J2*, *ATP5L*, and *ATP5S*, (**C**) *GH*, *GHR*, *IGF1*, *IGF1R*, *IGFBP2*, and *POU1F1*, and (**D**) *AMPKα2*, *AMPKβ2*, *AMPKγ3*, and *mTOR* in the skeletal muscle tissues of the chicken embryos at stage HH 20 exposed at different plasma potential for 4 min. Control and positive control were as described before. mRNA expression was determined by RT-PCR analysis, and normalized to the *β-actin* mRNA level. Values are expressed as the mean ± standard error (*n* = 3). *p < 0.05 versus control; **p < 0.01 versus control, according to one-way ANOVA and LSD test.

ATP levels in the skeletal muscle tissue of the dead embryos, for each of the different electrical potentials examined, decreased following exposure to plasma for 4 min and increases in the electric potential increased the inhibitory effects of the plasma on these levels. At 27.6 kV, exposure to plasma decreased the ATP concentration by 0.54-fold (*p <* 0.001; Figure 4A). *ATP* synthase mRNA expression levels in the skeletal muscle also correlated with plasma exposure potential. The greatest inhibitory effects were found at 27.6 kV, where 0.82-fold (*p <* 0.001) reductions in *ATP5A1*, 0.61-fold (*p <* 0.001) in *ATP5B*, 0.69-fold (*p <* 0.001) in *ATP5C1*, 0.75-fold (*p <* 0.001) in *ATP5F1*, 0.61-fold (*p <* 0.001) in *ATP5G1*, 0.54-fold (*p <* 0.001) in *ATP5G3*, 0.53-fold (*p <* 0.001) in *ATP5H*, 0.67-fold (*p <* 0.001) in *ATP5I*, 0.52-fold (*p <* 0.001) in *ATP5J*, 0.73-fold (*p <* 0.001) in *ATP5J2*, 0.69-fold (*p <* 0.001) in *ATP5L*, and 0.60-fold (*p <* 0.001) in *ATP5S* synthase mRNA expression were observed (Figure 4B). Similar decreases in mRNA expression in growth hormone (*GH*), growth hormone receptor (*GHR*), insulin-like growth factor 1 (*IGF1*), insulin-like growth factor 1 receptor (*IGF1R*), POU class 1 homeobox 1 (*POU1F1*), and mammalian target of rapamycin (*mTOR*) were found. At 27.6 kV, *GH*, *GHR*, *IGF1*, *IGF1R*, *POU1F1*, and *mTOR* expression decreased 0.79- (*p <* 0.001), 0.86- (*p <* 0.001), 0.70- (*p <* 0.001), 0.76- (*p <* 0.001), 0.56- (*p <* 0.001), and 0.74-fold (*p <* 0.001), respectively (Figure 4C and 4D). However, exposure to plasma incrementally increased the mRNA expression levels of insulin-like growth factor binding protein 2 (*IGFBP2*) and AMP-activated protein kinase (*AMPK*). At 27.6 kV, increases of 1.95- (*p <* 0.001), 1.15- (*p <* 0.001), 0.74- (*p <* 0.001), and 0.68-fold (*p <* 0.001) in the expression of *IGFBP2*, *AMPKα2*, *AMPKβ2*, and *AMPKγ3*, respectively, were observed (Figure 4C and 4D).

**Figure 5:**
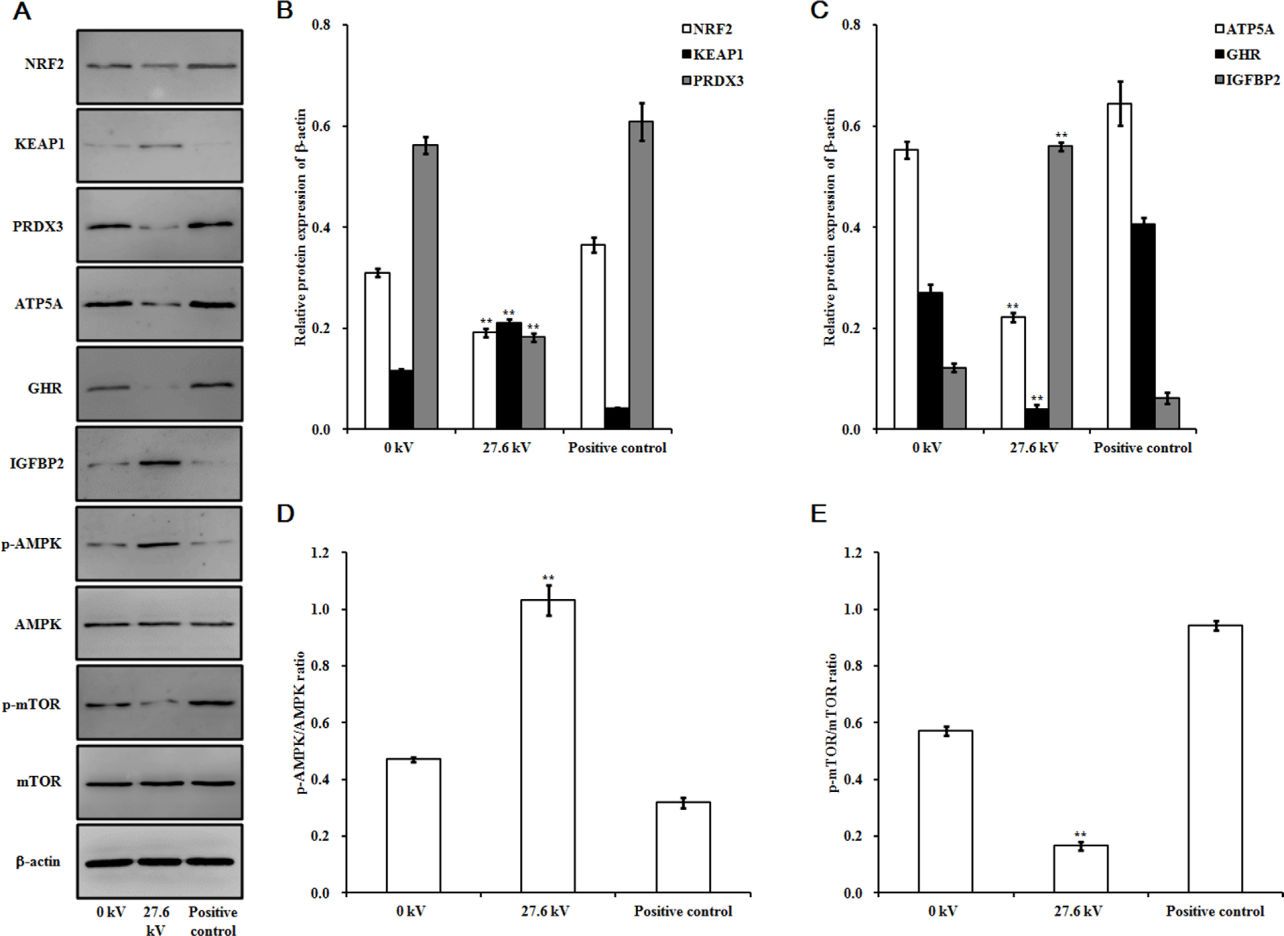
Effect of plasma on protein expression (**A**) Immunoblots of protein bands. Relative protein levels of (**B**) NRF2, KEAP1, and PRDX3, (**C**) ATP5A, GHR, and IGFBP2, (**D**) p-AMPK/AMPK, and (**E**) p-mTOR/mTOR. Protein was extracted from the skeletal muscle tissues of the chicken embryos at stage HH 20 exposed at 27.6 kV for 4 min. Control and positive control were as described before. The densitometric values of the NRF2, KEAP1, PRDX3, ATP5A, GHR, and IGFBP2 signals were normalized to the relevant β-actin signal. The densitometric value of each p-AMPK, AMPK, p-mTOR, and mTOR band was normalized to the β-actin signal in the same sample before calculating the p-AMPK/AMPK and p-mTOR/mTOR ratios. Values are expressed as the mean ± standard error (*n* = 3). *p < 0.05 versus control; **p < 0.01 versus control, according to one-way ANOVA and LSD test.

Plasma exposure also had significant effects on protein levels in the skeletal muscle tissue of the dead embryos. Exposure to plasma at 27.6 kV for 4 min significantly decreased the expressions of ATP5A and GHR, and reduced the phosphorylation level of mTOR in the dead embryos by 0.60- (*p* < 0.001), 0.85- (*p* < 0.001), and 0.71-fold (*p* < 0.001), respectively (Figure 5C and 5E), compared to that in control embryos. However, this same exposure to plasma increased IGFBP2 expression and the level of phosphorylation of AMPK by 3.58- (*p* < 0.001) and 1.19-fold (*p* < 0.001), respectively (Figure 5C and 5D).

## DISCUSSION

In a previous study, we showed exposure to non-thermal DBD plasma at certain electrical potentials and for specific durations can promote soybean sprout growth [9]. Therefore, this study explored the effects of plasma on chicken embryonic development. We chose to expose the embryos for 4 min to plasma, at different electrical potentials, in order to determine the effects on embryonic development. We examined the effects of non-thermal DBD plasma exposure on embryonic development by irradiating fertilized eggs at the HH 04 and HH 20 stages of development. Chicken embryos at stage HH 04 or HH 20 treated with plasma at 11.7 kV for 1 min were used as the positive control because this exposure condition improved chicken embryo growth. Our results show that plasma exposure at 11.7 kV for 1 min promoted chicken embryonic development, but 4 min of exposure resulted in dose-dependent embryonic death; at higher potentials (up to 27.6 kV) the rate of death increased. These results are consistent with previous findings that showed low-doses of plasma enhance cell proliferation, while high-doses induce cell death [1, 2, 18–20]. Furthermore, longer durations of plasma exposure increased apoptotic cell number in immune cell lines [15], decreased cell viability and proliferation [31], and caused endothelial cell toxicity [1]. These findings led us to conclude that 4 min exposure would be lethal to the chicken embryos.

Some early embryonic development takes place in fertilized eggs before incubation [11]. Once incubation begins, a pointed, thickened layer of cells becomes visible at the caudal end of the embryo. Embryos at stage HH 20 have all of the organs needed for growth [10]. Our results show that embryos at stage HH 20 have a longer survival time following exposure to plasma than the earlier stage HH 04 embryos. This is likely due to the establishment of a complete embryo structure by the end of stage HH 20, which would result in increased resistance to the harmful external factors. In addition, this is supported by the facts that embryos have stage-dependent resistances to various external factors during embryogenesis [12–14]. But we are not sure whether mammals have stage-dependent resistance to non-thermal DBD plasma because the embryonic development of mammals is different from that of chickens.

Non-thermal DBD plasma treatment induces oxidative stress from the diffusion of plasma-generated ROS, or from stimulation of the cell’s own ROS generating mechanisms [32], which results in decreased cell proliferation and differentiation [31], even cell death [29, 30, 32]. Our results show 4 min plasma exposure generated excessive ROS and increased MDA activity in dead embryos, which were mediated by decreasing cellular antioxidant activity and metabolic viability [20, 33], and correlated with elevated *NOX4* mRNA expression, which catalyzes the reduction of molecular oxygen to generate ROS [34–36]. NRF2 directly affects ROS levels by regulating the antioxidant defense systems through the induction of catabolism in SOD, CAT, GPx, and PRDX [26, 28, 37]. KEAP1 targets NRF2 for proteasomal degradation [27, 38, 39]. We found plasma increased KEAP1 level and decreased NRF2 level, activity and mRNA level of SOD, CAT, and GPx, and PRDX mRNA and protein in the dead embryos. These facts indicated oxidative stress was involved in the plasma-induced chicken embryonic death, resulting from the excessive accumulation of ROS and deficiency of antioxidant defense system that was suppressed by the disruption of NRF2-antioxidant signaling pathway.

ATP synthase is an important enzyme that provides energy for the cell via the production of ATP. Plasma treatment causes a decrease in mitochondrial transmembrane potential and subsequent mitochondrial enzymatic dysfunction and morphological alterations [40, 41] as a result of intracellular ROS accumulation that induces mitochondrial DNA oxidative damage, and disrupts DNA transcription and ATP production in the mitochondria [42]. Our results show exposure to plasma decreased ATP concentration, *ATP* synthase subunit mRNA level and ATP5A protein expression in the embryo skeletal muscle tissue. The results indicate that sustained plasma exposure inhibits ATP production, thus, negatively impacting cell proliferation and embryonic development. It is important to mention that ATP level in this study of plasma on chicken embryos was in contrast to other’s study that plasma-induced secretion of ATP and fluctuations in ATP level as a representative secreted damage-associated molecular patterns in carcinoma cells [32]. In addition, our results that plasma increased *AMPK* mRNA level and AMPK phosphorylation, decreased *mTOR* mRNA level and mTOR phosphorylation in dead embryo skeletal muscle tissue can be explained by the fact that ATP depletion activates AMPK, which in turn phosphorylates and activates the tuberous sclerosis complex, leading to the inhibition of mTOR [43].

We found that growth-related genes are involved in the plasma-treated chicken embryonic development. The results show plasma decreased mRNA level of *GH*, *GHR*, *IGF1*, *IGF1R*, and *POU1F1*, and increased *IGFBP2* mRNA and protein expression in the dead embryos. GH stimulates muscle and bone growth, and cell reproduction in animals [44] through its interaction with GHR [45]. GH-GHR binding stimulates the synthesis and release of IGF-1 [46], which is essential for embryonic development and post-hatching growth [47]. IGFBP2, the major IGFBP in chicken, has a specific, high-affinity for IGF1 and blocks IGF1 access to IGF1R, thereby inducing antiproliferative effects [47]. POU1F1 has a high affinity DNA binding domain which targets the genes that encode GH [48]. Our results indicate plasma exposure slows the signaling cascade involving GH and IGF-1, which induces the inhibition of cell reproduction and growth during embryonic development. In addition, GH has been shown to decrease the rate of mitochondrial ROS generation [49], and IGF-1 expression reduces ROS production from metabolism [50]. These previous findings may explain how the low mRNA level of *GH* and *IGF1* in the plasma-treated embryos contributed to increased intracellular ROS levels, and thereby, inhibiting embryonic development.

In conclusion, the duration and intensity of plasma exposure must be optimized for use in animal systems. Our results demonstrate the potential for non-thermal DBD plasma treatment in the promotion of growth in chicken, which we believe to be worthy of further exploration.

## MATERIALS AND METHODS

### Ethics

This study was carried out in strict accordance with the recommendations in the Guide for the Care and Use of Laboratory Animals of the National Institutes of Health (NIH Pub. No. 85–23, revised 1996). Animal handling protocols were approved by the Animal Care and Use Committee of Jeju National University (approval number: 2016–0022).

### Plasma treatment and fertilized egg incubation

**Figure 6:**
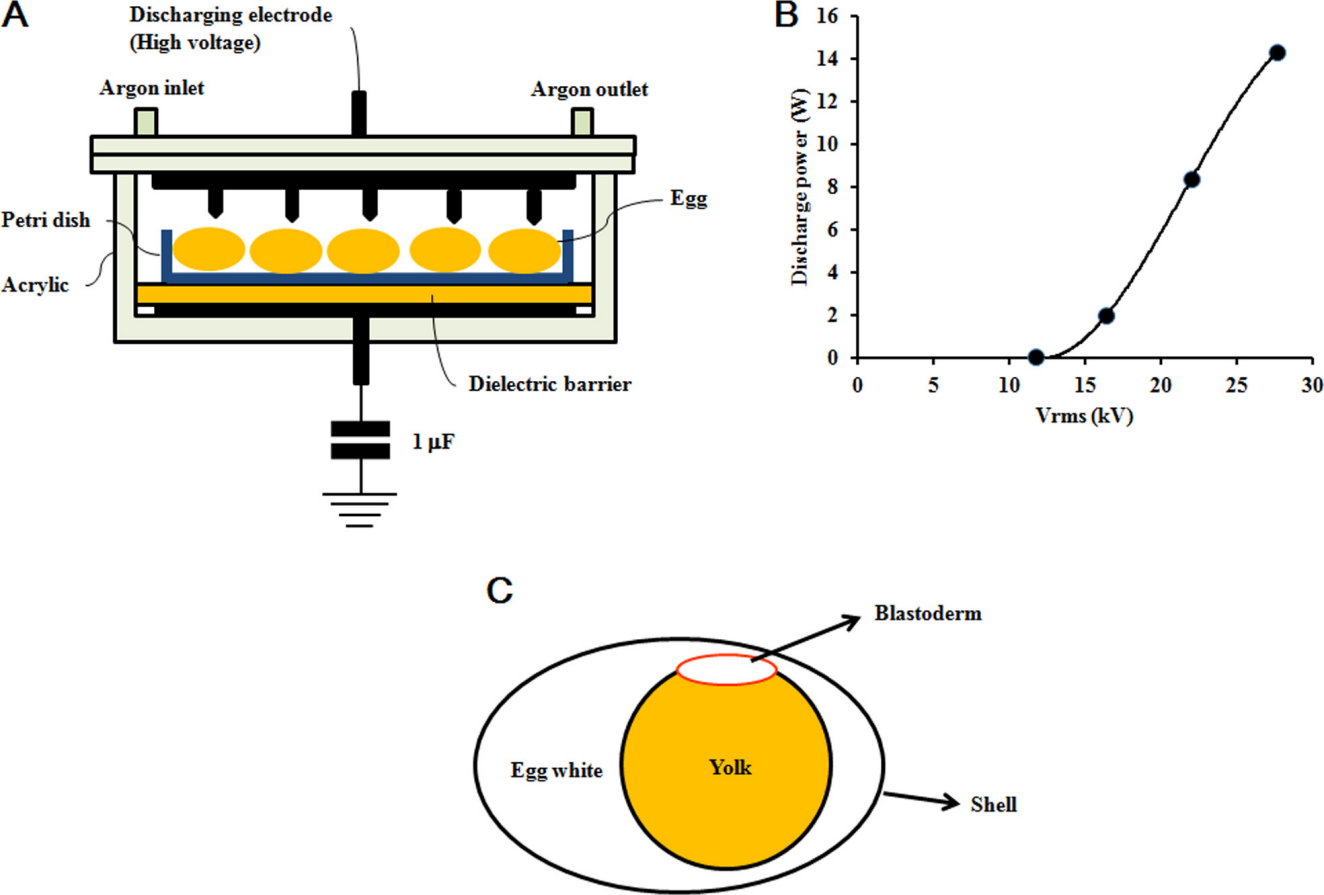
Non-thermal DBD plasma treatment (**A**) Plasma reactor schematic. (**B**) Voltage and discharge power applied in plasma treatment. (**C**) Fertilized egg schematic.

Figure 6A shows the dielectric barrier discharge plasma reactor, which operates at atmospheric pressure, used in this study. The reactor consists of two 100 mm wide disk-shaped electrodes and a 5 mm thick glass dielectric barrier. The upper electrode has 16 needles (thickness: 1 mm; length: 2.5 mm) that are evenly distributed on its inner surface, pointing downward towards the lower electrode. The distance between the needle tip and the ceramic dielectric barrier placed on the lower electrode is 30 mm. The plasma reactor was energized by high voltage alternating current (operating frequency: 60 Hz), and pure argon was fed to the reactor at a flow rate of 2 l/min. The voltage was measured using a digital oscilloscope (Tektronix, Beaverton, OR, USA) and a 1000:1 high voltage probe (P6015, Tektronix). The discharge power was determined using a voltage-charge Lissajous plot, and the charge was recorded by measuring the voltage across the 1.0 µF capacitor connected to the plasma reactor in series. As shown in Figure 6B, the discharge power increases exponentially from 0.05 to 14.28 W, as the voltage is changed from 11.7 to 27.6 kV rms.

Fresh fertilized eggs (approximately 50 g each) were obtained from farm-raised Hyline Brown hens (Jeju National University, Jeju, Republic of Korea). The hens were artificially inseminated twice a week with semen from Korean native cocks. The egg shells were wiped with 70% ethanol and put into an incubator with 45% to 65% relative humidity at 37.5°C and rotated 90 degrees every 2 hours. The embryos used in the plasma treatments were at either stage HH 04 or HH 20 in development. The embryos were placed in the plasma reactor with the blastoderm near the voltage probe (Figure 6C) and treated at different potentials for 4 min at room temperature, and returned to the incubator. Fertilized eggs not treated with plasma were used as the control group (0 kV). Embryos at stage HH 04 or HH 20 treated with plasma at 11.7 kV for 1 min were correspondingly used as the positive control group. Candling was performed on days 5, 12, and 19 during incubation. All dead embryos were removed from the shell by tearing the allantois and amnion with forceps. Eight fertilized eggs were used in each group. The number of dead embryos was recorded, and death day was estimated according to Hamburger-Hamilton stages [10].

### ROS, antioxidant enzyme, and ATP analyses

Skeletal muscle homogenates were prepared out in 1 ml ice-cold 0.1% sodium dodecyl sulfate (SDS; dissolved in PBS, 0.05 mol/l, pH 7.4) using a homogenizer, followed by centrifugation (12,000 × *g*) for 10 min at 4°C. Supernatant protein concentrations were analyzed using a Bicinchoninic Acid Protein Assay Kit (Sigma-Aldrich, St. Louis, MO, USA) and the concentrations were normalized using PBS. ROS levels were determined using the OxiSelect *In Vitro* ROS/RNS Assay Kit (Cell Biolabs, Inc., San Diego, CA, USA). The assay employs a proprietary quenched fluorogenic probe 2′,7′-dichlorodihydrofluorescein DiOxyQ (DCFH-DiOxyQ), which is a specific ROS/RNS probe that is based on similar chemistry to 2′,7′-dichlorodihydrofluorescein diacetate. The DCFH-DiOxyQ probe is first primed with a quench removal reagent, and subsequently stabilized in the highly reactive DCFH form, which is rapidly oxidized to the highly fluorescent 2′,7′-dichlorodihydrofluorescein (DCF) by ROS/RNS. The amount of DCF in the sample is determined based on the relative fluorescence units obtained using a DCF standard curve and the fluorescence intensity of DCF is proportional to the total ROS/RNS level within the sample, using a GloMax Discover Multimode Detection System fluorescence plate reader (Promega, Madison, WI, USA) at 480 nm excitation/530 nm emission.

The homogenate supernatant was assayed for SOD, CAT, GPx, MDA, and ATP concentrations using kits from Invitrogen and Sigma-Aldrich, according to the manufacturer’s instructions. The optical density of SOD, CAT, GPx, and MDA were detected using the GloMax Discover Multimode Detection System (Promega). The relative light unit values of ATP level were measured using a luminometer (Sirius L Tube Luminometer, Titertek Berthold, Germany). SOD activity was expressed as U/mg protein, where one unit was defined as the amount of enzyme that reduces 1.0 µmole of superoxide to molecular oxygen and hydrogen peroxide per min at 25°C. CAT activity was expressed as U/mg protein, wher U was defined as the amount of enzyme that decomposes 1.0 µmole of hydrogen peroxide to oxygen and water per min at 25°C. GPx activity was expressed as mU/mg protein, where mU was defined as the amount of enzyme that will cause the oxidation of 1.0 nmole of NADPH to NADP^+^ under the assay kit condition per min at 25 °C. MDA and ATP levels were expressed as nmole/mg protein in muscle tissue.

### RT-PCR analysis

Total RNA was extracted and purified from chicken embryo skeletal muscle tissue using the TRIzol Reagent (Invitrogen, Thermo Fisher Scientific, Waltham, MA, USA), following the manufacturer’s instructions. RT-PCR analysis was performed using SuperScript III First-Strand Synthesis System for RT-PCR (Invitrogen), Prime Taq Premix (2×) (GENETBIO, Yuseong-gu, Daejeon, South Korea), EvaGreen Dye (Biotium, Hayward, CA, USA). First-strand cDNA synthesis was performed using 1 μl total RNA (1 μg/μl), following the manufacturer’s protocol. RT-PCR was performed using a StepOne Real-time PCR system (Applied Biosystems, Thermo Fisher Scientific). The RT-PCR conditions included initial denaturation at 95°C for 10 min, followed by denaturation at 95°C for 15 s and annealing for 1 min at the temperatures shown in Table 2 for 40 cycles, and melting curves were derived at temperatures of 95°C for 15 s, 60°C for 1 min, and increased to 95°C for 15 s by 0.3°C. Primer sequences for RT-PCR ware shown in Table 2.

**Table 2:** Primer sequences for the RT-PCR

Gene	Sequence number	Sequence position	Product length (bp)	Annealing Temperature (°C)	Sequence (5′ to 3′)
β-actin	NM_205518.1	625–818	194	57	F: GTGCGTGACATCAAGGAGAAGC
					R: CCACAGGACTCCATACCCAAGA
NOX4	NM_001101829.1	28–157	130	57	F: CGAGGATCTCAGAAGGTTGC
					R: GAGCATTCACCAGATGAGCA
NRF2	NM_205117.1	484–619	136	57	F: AAAACGCTGAACCACCAATC
					R: GCTGGAGAAGCCTCATTGTC
KEAP1	KU321503.1	1227–1485	259	57	F: GTATCACAGCAGCGTGGAGA
					R: GGCGTAGATGCAGTTGTTGA
SOD	NM_205064.1	106–278	173	55	F: ATTACCGGCTTGTCTGATGG
					R: CCTCCCTTTGCAGTCACATT
CAT	NM_001031215.2	1067–1276	210	55	F: CTCATTCCAGTGGGCAAGAT
					R: GTAGGGGCAATTCACAGGAA
GPx	NM_001277853.2	353–474	122	55	F: ATGTTCGAGAAGTGCGAGGT
					R: ATGATGTACTGCGGGTTGGT
PRDX1	NM_001271932.1	358–545	188	56	F: ACAAGGTGGTTTGGGCACTA
					R: TCTCATCAACAGAACGGCCA
PRDX3	XM_426543.5	414–551	138	56	F: TTTCACCTTTGTGTGCCCCA
					R: TTGCGCGGGGTATTTATCCA
PRDX4	XM_001233999.3	595–733	139	56	F: TGCACTTAGGGGCCTTTTCA
					R: TTCTCCATGCTTGTCCGTGT
PRDX6	NM_001039329.2	189–340	152	58	F: TGAGTTCAGCAAACGCAACG
					R: GCTCTCGGTCCTTATCAGCG
ATP5A1	NM_204286.1	1207–1364	158	57	F: GGTATCCGTCCAGCCATCAA
					R: GCATCCAAATCAGACCCAAACT
ATP5B	NM_001031391.2	482–637	156	57	F: GCCCCATCACAACGAAACAG
					R: CGCCTCCAAACAAACCAATC
ATP5C1	NM_001278096.1	272–411	140	57	F: ATTAAGGCACCCGAGGACAA
					R: ACTTCCTTCCCTGCATTGGA
ATP5F1	XM_417993.4	437–644	208	57	F: CATTGGAGACTGCCATTGAGG
					R: TGATCTTGCTCTTTCTGACGCTT
ATP5G1	XM_001233602.3	287–536	250	57	F: CAGGAGCAGGTATTGGGACA
					R: TTGTCAGTCTGGAACGCTCT
ATP5G3	NM_001277855.1	141–288	148	57	F: CCAAAACGCTGTCTCCCAAC
					R: ACCGAAGACCGTTCCAATACC
ATP5H	XM_001232598.3	332–551	220	57	F: CTGAAGGTCCCTGAACCAGT
					R: ACTTCTCCCTGTCCAGTCTG
ATP5I	NM_001097534.2	74–240	167	57	F: TCTCGCCCCTCATCAAGTTC
					R: TGCCAGTTCCTTTGCAATCC
ATP5J	XM_004938370.1	58–197	140	58	F: CACTTGCGGAGAAACATCGGT
					R: CCTACATCAACAGGTCCTCCAGC
ATP5J2	NM_001257200.1	170–263	94	57	F: GCCTCGGTGGTATCAGTATGGT
					R: TACTTCCTGCGGCGGTCAT
ATP5L	XM_015298211	250–377	128	57	F: CCATGGTCAGGAGCTTTCAG
					R: GCCTCGTTTGCCTATGATCTC
ATP5S	NM_001277562.1	46–279	234	57	F: TCCCCTTCCCCTTTCTTTCC
					R: CATAGCCTTGATAGCGCACC
GH	NM_204359.2	104–284	181	57	F: TGTTTGCCAACGCTGTGCT
					R: TTCTGCTGGGCGTCATCCT
GHR	NM_001001293.1	1070–1299	230	57	F: GTCACACAGTTGCTTGGGAG
					R: TATGCGGCTGTTGGGTATCT
IGF1	NM_001004384.2	188–316	129	58	F: AGTTCGTATGTGGAGACAGAGGC
					R: CCAGCCTCCTCAGGTCACAAC
IGF1R	NM_205032.1	2961–3114	154	57	F: TTGTGCTCCCCATTGCTTTC
					R: GGAACGTACACATCCGAAGC
IGFBP2	NM_205359.1	582–793	212	57	F: TCACAACCACGAGGACTCAAAG
					R: GCTGCCCATTCACCGACAT
POU1F1	NM_204319.1	560–754	195	57	F: ATGTTGGCGAAGCACTGGC
					R: GCTTCCTCTTCCGCTCATTCA
AMPKα2	NM_001039605.1	726–943	218	57	F: GGAGGCGTGTTTTACATCCC
					R: AACTTCTCACAGACCTCCCG
AMPKβ2	NM_001044662.1	435–661	227	57	F: CCAGTGTTTTCAGCTCCCAC
					R: GAGGTCCAGGATAGCGACAA
AMPKγ3	NM_001031258.2	183–320	138	57	F: GCTGGAACCCGACAACAATT
					R: GCCTTCTTGATCTCCAGGGT
mTOR	XM_417614.4	119–309	191	57	F: TGAAGGGGTCAAGGCAATCC
					R: GGCGAGCAGTGGTTGTGGAT

NOX4, nicotinamide adenine dinucleotide phosphate oxidase 4; NRF2, nuclear factor erythroid 2-related factor 2; KEAP1, kelch like ECH associated protein 1; SOD, superoxide dismutase; CAT, catalase; GPx, glutathione peroxidase; PRDX, peroxiredoxin; ATP5A1, ATP synthase, H+ transporting, mitochondrial F1 complex, alpha subunit 1; ATP5B, ATP synthase, H+ transporting, mitochondrial F1 complex, beta polypeptide; ATP5C1, ATP synthase, H+ transporting, mitochondrial F1 complex, gamma polypeptide 1; ATP5F1, ATP synthase, H+ transporting, mitochondrial Fo complex, subunit B1; ATP5G1, ATP synthase, H+ transporting, mitochondrial Fo complex, subunit C1; ATP5G3, ATP synthase, H+ transporting, mitochondrial Fo complex, subunit C3; ATP5H, ATP synthase, H+ transporting, mitochondrial Fo complex, subunit D; ATP5I, ATP synthase, H+ transporting, mitochondrial Fo complex, subunit E; ATP5J, ATP synthase, H+ transporting, mitochondrial Fo complex, subunit F6; ATP5J2, ATP synthase, H+ transporting, mitochondrial Fo complex, subunit F2; ATP5L, ATP synthase, H+ transporting, mitochondrial Fo complex, subunit G; ATP5S, ATP synthase, H+ transporting, mitochondrial Fo complex, subunit S; GH, growth hormone; GHR, growth hormone receptor; IGF1, insulin-like growth factor 1; IGF1R, insulin-like growth factor 1 receptor; IGFBP2, insulin-like growth factor binding protein 2; POU1F1, POU class 1 homeobox 1; AMPKα2, AMP-activated protein kinase catalytic subunit alpha 2; AMPKβ2, AMP-activated protein kinase non-catalytic subunit beta 2; AMPKγ3, AMP-activated protein kinase non-catalytic subunit gamma 3; mTOR, mammalian target of rapamycin.

The cycle threshold values for each sample were determined using triplicate measurements. Equivalent dilutions were calculated using a standard curve and normalized to the housekeeping gene (β*-actin*). Reaction product sequences were confirmed by direct nucleotide sequencing using an ABI PRISM 7700 Sequence Detector (Applied Biosystems). Relative expression levels were calculated using the 2^-ΔΔCT^ method.

### Western blotting

For extraction of total protein, 0.3 g chicken skeletal muscle was cut into small pieces and resuspended in lysis buffer (25 mM Tris-HCl, pH 7.6, 150 mM NaCl, 1% NP-40, 1% sodium deoxycholate, 0.1% SDS, 1 mM phenylmethylsulfonyl fluoride, and 1 mg/l chymostatin) at 4 °C for 30 min prior to centrifugation for 10 min at 11,000 × *g*. The protein concentrations were determined using a Bicinchoninic Acid Protein Assay Kit (Sigma-Aldrich, St. Louis, MO, USA) using bovine serum albumin (BSA) as a standard protein. Proteins were separated by 12% sodium dodecyl sulfate-polyacrylamide gel electrophoresis and transferred to polyvinylidene fluoride membranes by wet electrophoretic transfer (Bio-Rad, Hercules, California, USA). After blocking in PBS-Tween containing 5% dried skimmed milk or 3% BSA for 2 h at room temperature, membranes were incubated with primary detection antibodies raised against NRF2 (mouse, 1:200; Santa Cruz Biotechnology, Dallas, Texas, USA), KEAP1 (mouse, 1:200; Santa Cruz Biotechnology), PRDX3 (mouse, 1:200; Santa Cruz Biotechnology), ATP5A (rabbit, 1:250; Abcam, Cambridge, UK), GHR (rabbit, 1:500; Bioss, Woburn, MA, USA), IGFBP2 (rabbit, 1:500; Bioss), phosphorylated AMPK (Thr172, p-AMPK; rabbit, 1:1,000; Cell Signaling Technology, Beverly, MA, USA), AMPK (rabbit, 1:1,000; Cell Signaling Technology), phosphorylated mTOR (Ser2448, p-mTOR; rabbit, 1:1,000; Cell Signaling Technology), mTOR (rabbit, 1:1,000; Cell Signaling Technology), and β-actin (rabbit, 1:1,000; Bioss) at 4°C overnight. After incubation with goat anti-rabbit immunoglobulin G coupled to horseradish peroxidase (1:5,000; Abcam) or goat anti-mouse secondary antibody (1:5,000; Abcam) for 2 h at room temperature, proteins were visualized by enhanced chemiluminescence (SuperSignal West Pico; Thermo Fisher Scientific). Band intensities were quantified using Quantity One software (Bio-Rad). The densitometric values of the NRF2, KEAP1, PRDX3, ATP5A, GHR, and IGFBP2 signals were normalized to the relevant β-actin signal. The densitometric value of each p-AMPK, AMPK, p-mTOR, and mTOR band was normalized to the β-actin signal in the same sample before calculating the p-AMPK/AMPK and p-mTOR/mTOR ratios.

### Statistical analysis

All experiments were performed in triplicate. Values are expressed as the mean ± standard error. Statistical analyses were performed using the Statistical Package for the Social Sciences (SPSS version 16.0; SPSS, Chicago, IL, USA). Data were analyzed by one-way ANOVA and Fisher’s least significant difference (LSD) test to determine treatment differences.

## Abbreviations

ATP: adenosine triphosphate
AMPK: AMP-activated protein kinase
BSA: bovine serum albumin
CAT: catalase
DBD: dielectric barrier discharge
GPx: glutathione peroxidase
GH: growth hormone
GHR: growth hormone receptor
H_2_O_2_: hydrogen peroxide
IGF1: insulin-like growth factor 1
IGF1R: insulin-like growth factor 1 receptor
IGFBP2: insulin-like growth factor binding protein 2
KEAP1: kelch like ECH associated protein 1
LSD: least significant difference
MDA: malondialdehyde
mTOR: mammalian target of rapamycin
NOX4: nicotinamide adenine dinucleotide phosphate oxidase 4
NRF2: nuclear factor erythroid 2-related factor 2
POU1F1: POU class 1 homeobox 1
PRDX: peroxiredoxins
ROS: reactive oxygen species
SOD: superoxide dismutase
SDS: sodium dodecyl sulfate

## References

[1] Kalghatgi S, Friedman G, Fridman A, Clyne AM (2010). Endothelial cell proliferation is enhanced by low dose non-thermal plasma through fibroblast growth factor-2 release. Ann Biomed Eng.

[2] Kalghatgi S, Kelly CM, Cerchar E, Torabi B, Alekseev O, Fridman A, Friedman G, Azizkhan-Clifford J (2011). Effects of non-thermal plasma on mammalian cells. PLoS One.

[3] Kuchenbecker M, Bibinov N, Kaemlimg A, Wandke D, Awakowicz P, Viol W (2009). Characterization of DBD plasma source for biomedical applications. J Phys D Appl Phys.

[4] Justan I, Cernohorska L, Dvorak Z, Slavicek P (2014). Plasma discharge and time-dependence of its effect to bacteria. Folia Microbiol (Praha).

[5] Leduc M, Guay D, Leask RL, Coulombe S (2009). Cell permeabilization using a non-thermal plasma. New J Phys.

[6] Beate H, Thomas von W, Klaus-Dieter W, Ulrike L (2014). Non-thermal atmospheric-pressure plasma possible application in wound healing. Biomol Ther (Seoul).

[7] Fridman G, Peddinghaus M, Ayan H, Fridman A, Balasubramanian M, Gutsol A, Brooks A, Friedman G (2006). Blood coagulation and living tissue sterilization by floating-electrode dielectric barrier discharge in air. Plasma Chemistry and Plasma Processing.

[8] Kim CH, Bahn JH, Lee SH, Kim GY, Jun SI, Lee K, Baek SJ (2010). Induction of cell growth arrest by atmospheric non-thermal plasma in colorectal cancer cells. J Biotechnol.

[9] Zhang JJ, Jo JO, Huynh DL, Mongre RK, Ghosh M, Singh AK, Lee SB, Mok YS, Hyuk P, Jeong DK (2017). Growth-inducing effects of argon plasma on soybean sprouts via the regulation of demethylation levels of energy metabolism-related genes. Sci Rep.

[10] Hamburger V, Hamilton HL (1992). A series of normal stages in the development of the chick embryo. 1951. Dev Dyn.

[11] Patten BM (1920). The Early Embryology of the Chick.

[12] Zusman I, Ornoy A (1990). Embryonic resistance to chemical and physical factors: manifestation, mechanism, role in reproduction and in adaptation to ecology. Biol Rev Camb Philos Soc.

[13] Kavlock RJ, Daston GP (1997). Drug toxicity in embryonic development I: advances in understanding mechanisms of birth defects: morphogenesis and processes at risk.

[14] Hu LB, Lucio B, Schat KA (1993). Abrogation of age-related resistance to chicken infectious anemia by embryonal bursectomy. Avian Dis.

[15] Haertel B, Volkmann F, von Woedtke T, Lindequist U (2012). Differential sensitivity of lymphocyte subpopulations to non-thermal atmospheric-pressure plasma. Immunobiology.

[16] Keidar M, Walk R, Shashurin A, Srinivasan P, Sandler A, Dasgupta S, Ravi R, Guerrero-Preston R, Trink B (2011). Cold plasma selectivity and the possibility of a paradigm shift in cancer therapy. Br J Cancer.

[17] Zirnheld JL, Zucker SN, Disanto TM, Berezney R, Etemadi K (2010). Nonthermal plasma needle: development and targeting of melanoma cells. IEEE Trans Plasma Sci.

[18] Kieft IE, Kurdi M, Stoffels E (2006). Reattachment and apoptosis after plasma-needle treatment of cultured cells. IEEE Trans Plasma Sci.

[19] Nakai N, Fujita R, Kawano F, Takahashi K, Ohira T, Shibaguchi T, Nakata K, Ohira Y (2014). Retardation of C2C12 myoblast cell proliferation by exposure to low-temperature atmospheric plasma. J Physiol Sci.

[20] Kaushik NK, Kaushik N, Park D, Choi EH (2014). Altered antioxidant system stimulates dielectric barrier discharge plasma-induced cell death for solid tumor cell treatment. PLoS One.

[21] Fridman A, Chirokov A, Gutsol A (2005). Non-thermal atmospheric pressure discharges. J Phys D Appl Phys.

[22] Fridman G, Friedman G, Gutsol A, Shekhter AB, Vasilets VN, Fridman A (2008). Applied plasma medicine. Plasma Processes and Polymers.

[23] Ji AR, Ku SY, Cho MS, Kim YY, Kim YJ, Oh SK, Kim SH, Moon SY, Choi YM (2010). Reactive oxygen species enhance differentiation of human embryonic stem cells into mesendodermal lineage. Exp Mol Med.

[24] Arjunan KP, Friedman G, Fridman A, Clyne AM (2012). Non-thermal dielectric barrier discharge plasma induces angiogenesis through reactive oxygen species. J R Soc Interface.

[25] Kansanen E, Kuosmanen SM, Leinonen H, Levonen AL (2013). The Keap1-Nrf2 pathway: mechanisms of activation and dysregulation in cancer. Redox Biol.

[26] Nguyen T, Nioi P, Pickett CB (2009). The Nrf2-Antioxidant Response Element Signaling Pathway and Its Activation by Oxidative Stress. J Biol Chem.

[27] Li N, Alam J, Venkatesan MI, Eiguren-Fernandez A, Schmitz D, Di Stefano E, Slaughter N, Killeen E, Wang X, Huang A, Wang M, Miguel AH, Cho A (2004). Nrf2 Is a key transcription factor that regulates antioxidant defense in macrophages and epithelial cells: protecting against the proinflammatory and oxidizing effects of diesel exhaust chemicals. J Immunol.

[28] Ma Q (2013). Role of Nrf2 in Oxidative Stress and Toxicity. Annu Rev Pharmacol Toxicol.

[29] Blackert S, Haertel B, Wende K, von Woedtke T, Lindequist U (2013). Influence of non-thermal atmospheric pressure plasma on cellular structures and processes in human keratinocytes (HaCaT). J Dermatol Sci.

[30] Ma Y, Ha CS, Hwang SW, Lee HJ, Kim GC, Lee KW, Song K (2014). Non-thermal atmospheric pressure plasma preferentially induces apoptosis in p53-mutated cancer cells by activating ROS stress-response pathways. PLoS One.

[31] Balzer J, Heuer K, Demir E, Hoffmanns MA, Baldus S, Fuchs PC, Awakowicz P, Suschek CV, Opländer C (2015). Non-thermal dielectric barrier discharge (DBD) effects on proliferation and differentiation of human fibroblasts are primary mediated by hydrogen peroxide. PLoS One.

[32] Lin A, Truong B, Patel S, Kaushik N, Choi EH, Fridman G, Fridman A, Miller V (2017). Nanosecond-pulsed DBD plasma-generated reactive oxygen species trigger immunogenic cell death in A549 lung carcinoma cells through intracellular oxidative stress. Int J Mol Sci.

[33] Yan X, Xiong ZL, Zou F, Zhao SS, Lu XP, Yang GX, He GY, Ostrikov K (2012). Plasma-induced death of HepG2 cancer cells: intracellular effects of reactive species. Plasma Processes and Polymers.

[34] Schröder K, Zhang M, Benkhoff S, Mieth A, Pliquett R, Kosowski J, Kruse C, Luedike P, Michaelis UR, Weissmann N, Dimmeler S, Shah AM, Brandes RP (2012). Nox4 is a protective reactive oxygen species generating vascular NADPH oxidase. Circ Res.

[35] Kuroda J, Ago T, Matsushima S, Zhai P, Schneider MD, Sadoshima J (2010). NADPH oxidase 4 (Nox4) is a major source of oxidative stress in the failing heart. Pro Nat Acad Sci USA.

[36] Ershova ES, Sergeeva VA, Chausheva AI, Zheglo DG, Nikitina VA, Smirnova TD, Kameneva LV, Porokhovnik LN, Kutsev SI, Troshin PA, Voronov II, Khakina EA, Veiko NN (2016). Toxic and DNA damaging effects of a functionalized fullerene in human embryonic lung fibroblasts. Mutat Res Genet Toxicol Environ Mutagen.

[37] Cullinan SB, Zhang D, Hannink M, Arvisais E, Kaufman RJ, Diehl JA (2003). Nrf2 is a direct PERK substrate and effector of PERK-dependent cell survival. Mol Cell Biol.

[38] Schieber M, Chandel NS (2014). ROS function in redox signaling and oxidative stress. Curr Biol.

[39] Jaramillo MC, Zhang DD (2013). The emerging role of the Nrf2–Keap1 signaling pathway in cancer. Genes Dev.

[40] Ahn HJ, Kim KI, Kim G, Moon E, Yang SS, Lee JS (2011). Atmospheric-pressure plasma jet induces apoptosis involving mitochondria via generation of free radicals. PLoS One.

[41] Panngom K, Baik KY, Nam MK, Han JH, Rhim H, Choi EH (2013). Preferential killing of human lung cancer cell lines with mitochondrial dysfunction by nonthermal dielectric barrier discharge plasma. Cell Death Dis.

[42] Clayton DA (1984). Transcription of the mammalian mitochondrial genome. Annu Rev Biochem.

[43] Schneider A, Younis RH, Gutkind JS (2008). Hypoxia-induced energy stress inhibits the mTOR pathway by activating an AMPK/REDD1 signaling axis in head and neck squamous cell carcinoma. Neoplasia.

[44] Veldhuis JD, Roemmich JN, Richmond EJ, Rogol AD, Lovejoy JC, Sheffield-Moore M, Mauras N, Bowers CY (2005). Endocrine control of body composition in infancy, childhood, and puberty. Endocr Rev.

[45] Gonzalez L, Curto LM, Miquet JG, Bartke A, Turyn D, Sotelo AI (2007). Differential regulation of membrane associated-growth hormone binding protein (MA-GHBP) and growth hormone receptor (GHR) expression by growth hormone (GH) in mouse liver. Growth Horm IGF Res.

[46] Van Vught AJ, Nieuwenhuizen AG, Brummer RJ, Westerterp-Plantenga MS (2008). Effects of oral ingestion of amino acids and proteins on the somatotropic axis. J Clin Endocrinol Metab.

[47] Kita K, Nagao K, Okumura J (2005). Nutritional and tissue specificity of IGF-I and IGFBP-2 gene expression in growing chickens. Asian Australas J Anim Sci.

[48] Sobrier ML, Tsai YC, Perez C, Leheup B, Bouceba T, Duquesnoy P, Copin B, Sizova D, Penzo A, Stanger BZ, Cooke NE, Liebhaber SA, Amselem S (2016). Functional characterization of a human POU1F1 mutation associated with isolated growth hormone deficiency: a novel etiology for IGHD. Hum Mol Genet.

[49] Sanz A, Gredilla R, Pamplona R, Portero-Otin M, Vara E, Tresguerres JA, Barja G (2005). Effect of insulin and growth hormone on rat heart and liver oxidative stress in control and caloric restricted animals. Biogerontology.

[50] Gomes-Marcondes MC, Tisdale MJ (2002). Induction of protein catabolism and the ubiquitin-proteasome pathway by mild oxidative stress. Cancer Lett.

